# Mathematical Assessment of Machine Learning Models Used for Brain Tumor Diagnosis

**DOI:** 10.3390/diagnostics13040618

**Published:** 2023-02-08

**Authors:** Dilber Uzun Ozsahin, Efe Precious Onakpojeruo, Berna Uzun, Mubarak Taiwo Mustapha, Ilker Ozsahin

**Affiliations:** 1 Department of Medical Diagnostic Imaging, College of Health Science, University of Sharjah, Sharjah 27272, United Arab Emirates; 2 Operational Research Centre in Healthcare, Near East University, TRNC Mersin 10, Nicosia 99138, Turkey; 3 Department of Statistics, Carlos III University of Madrid, 28903 Madrid, Spain; 4 Department of Mathematics, Near East University, TRNC Mersin 10, Nicosia 99138, Turkey; 5 Brain Health Imaging Institute, Department of Radiology, Weill Cornell Medicine, New York, NY 10065, USA

**Keywords:** brain tumors diagnosis, magnetic resonance imaging, machine learning, convolutional neural networks, fuzzy PROMETHEE, decision making

## Abstract

The brain is an intrinsic and complicated component of human anatomy. It is a collection of connective tissues and nerve cells that regulate the principal actions of the entire body. Brain tumor cancer is a serious mortality factor and a highly intractable disease. Even though brain tumors are not considered a fundamental cause of cancer deaths worldwide, about 40% of other cancer types are metastasized to the brain and transform into brain tumors. Computer-aided devices for diagnosis through magnetic resonance imaging (MRI) have remained the gold standard for the diagnosis of brain tumors, but this conventional method has been greatly challenged with inefficiencies and drawbacks related to the late detection of brain tumors, high risk in biopsy procedures, and low specificity. To circumvent these underlying hurdles, machine learning models have recently been developed to enhance computer-aided diagnosis tools for advanced, precise, and automatic early detection of brain tumors. This study takes a novel approach to evaluate machine learning models (support vector machine (SVM), random forest (RF), gradient-boosting model (GBM), convolutional neural network (CNN), K-nearest neighbor (KNN), AlexNet, GoogLeNet, CNN VGG19, and CapsNet) used for the early detection and classification of brain tumors by deploying the multicriteria decision-making method called fuzzy preference ranking organization method for enrichment evaluations (PROMETHEE), based on selected parameters, in this study: prediction accuracy, precision, specificity, recall, processing time, and sensitivity. To validate the results of our proposed approach, we performed a sensitivity analysis and cross-checking analysis with the PROMETHEE model. The CNN model, with an outranking net flow of 0.0251, is considered the most favorable model for the early detection of brain tumors. The KNN model, with a net flow of −0.0154, is the least appealing option. The findings of this study support the applicability of the proposed approach for making optimal choices regarding the selection of machine learning models. The decision maker is thus afforded the opportunity to expand the range of considerations which they must rely on in selecting the preferred models for early detection of brain tumors.

## 1. Introduction

The practice of identifying a disease from its signs and symptoms has gained prominence, leading to significant strides in therapeutic measures [1]. Disease diagnosis still relies on physical and chemical examinations [2,3,4]. Artificial intelligence (AI) models can be used to assess [5], diagnose, and plan the pre-operative stages of brain tumor cancer [6]. Brain tumors are a serious mortality factor [5], and their detection is often difficult due to their heterogeneous nature [7]. In recent decades, brain tumor occurrences have been on the rise, resulting in increased cases of mortality [5,7]. The brain is a complex component of the human anatomy [8], and once symptoms of tumors appear, it becomes too late to treat them [5,6]. Computer-aided devices for diagnosis, such as magnetic resonance imaging (MRI), have been assistive in the detection of brain tumors [7]. In the USA, about 700,000 people were living with a brain tumor in 2021 [9]. Brain tumors can be benign or malignant [5,10], with malignant tumors capable of spreading beyond the brain region [5,7,8]. A study [7] reported that brain tumors can occur in both adults and children in stages and grades. The major causes of brain tumors are cancer-related diseases and morbidity factors [8]. Computer-aided devices for diagnosis through MRI have remained the gold standard [6] for the diagnosis of brain tumors [7], but recent breakthroughs in early detection have significantly lowered brain tumor cancer mortality rate and related morbidity factors [10,11]. Some researchers have deployed deep learning models to improve the diagnosis of brain tumors.

In recent years, artificial intelligence (AI) has been used to improve the diagnosis of brain tumors. One of the main ways that AI is being used is through deep learning (DL) models [12]. These models are trained on large amounts of data and can then be used to identify patterns and features that are not easily visible to radiologists. This can be particularly useful for identifying early-stage brain tumors, which are often difficult to detect using traditional imaging techniques [6].

One of the main advantages of using AI for brain tumor diagnosis is that it can help to improve the accuracy of diagnoses [13,14]. This is because AI models can analyze large amounts of data and identify patterns that may not be apparent to human radiologists. This can lead to more accurate and earlier diagnoses, which can improve patient outcomes. Another advantage of using AI for brain tumor diagnosis is that it can help to reduce the workload of radiologists. This is because AI models can automatically analyze images and identify areas of concern, leaving radiologists with more time to focus on other tasks.

Despite the potential benefits of using AI for brain tumor diagnosis, there are also some potential drawbacks to consider. One of the main concerns is that AI models may not be as accurate as human radiologists, particularly when it comes to identifying early-stage brain tumors. Additionally, AI models may not be able to consider certain patient-specific factors that could influence a diagnosis.

Model evaluations have always been based on performance evaluation metrics such as accuracy, F1 score, log loss, precision, recall, AUC, specificity, and so on. None have added important metrics to create more robust, adaptable, and compromising metrics. Recently, decision makers have sought a more robust method of incorporating additional metrics into the model selection process. Thus, several questions have arisen, such as what happens if a decision maker needs considerable critical factors that are not covered by the usual performance evaluation metrics? What happens if the accuracy of the model is essential to the decision makers? What if the decision maker is most concerned with the preferred model for classification and segmentation without having to undergo the process of deplorability before deciding? All of these questions are yet to be resolved and, as a result, a research gap has emerged.

This study takes another novel approach to evaluating machine learning models used in the early detection of brain tumors by deploying the MCDM method called fuzzy PROMETHEE to rank models based on more compromising selected weighted criteria from an expert’s point of view. A similar methodology has only been deployed once in a study by Mustapha et al. [1] in the classification and early detection of breast cancer. It has never been implemented in any brain tumor study, except for [15], which deployed fuzzy PROMETHEE to evaluate treatment techniques for brain cancer. Therefore, this method is unique in its kind to this study.

## 2. Methods

### 2.1. Multi-Criteria Decision-Making Method (MCDM)

MCDM methods are one of the most significant ways to determine the best decision among several alternatives. It contains effective tools with enormous potential in the field of artificial intelligence, including how to use various criteria to compare a set of alternatives [16]. MCDM is a technique that combines various, and at times competing, qualitative or quantitative criteria, and yields a solution that calls for agreement [17]. Choosing the optimum alternative/s using several MCDM approaches has been suggested in recent years. It draws on knowledge from a variety of disciplines, including cognitive and behavioral decision theory, computer science, economics, data management, information systems, mathematics, and decision analysis applied to the field of environmental health [18]. In numerous application domains since the late 1950s, numerous MCDM methods and approaches have been created, put forth, and successfully implemented. The goal of MCDM is to help decision makers choose the optimum alternative/s that satisfy their requirements and are in line with their desired preferences [17,18,19]. 

There are numerous MCDM methods available, including the analytic hierarchy process (AHP), a technique for order of preference by similarities to ideal solution (TOPSIS), elimination et Choix traduisant la réalité (ELECTRE), preference ranking organization method for enriching evaluations (PROMETHEE), visekriterijumska optimizcija i kaompromisno resenje (VIKOR), and data envelopment analysis (DEA). It is difficult to determine the best method, since each has its own benefits and disadvantages. 

### 2.2. Application of Fuzzy PROMETHEE to the Study

The PROMETHEE method is an MCDM tool that enables users to evaluate and rank alternatives in accordance with specified criteria [20]. Brans and Vince developed the PROMETHEE method in 1985 [21] to compare options based on the chosen criteria. The PROMETHEE method gives the user total control over the preference values of the criteria [1,15]. PROMETHEE is one of the widely used decision-making tools in a variety of fields [22]. The weights assigned to the specified criteria and the preference function are the only information needed from the decision maker to determine how much better the alternative is on each criterion. The PROMETHEE method still cannot handle fuzzy data in a real-world decision-making setting; hence, the hybrid usage of fuzzy logic with the PROMETHEE method enables the decision maker to analyze the data under uncertainty.

In its most basic form, fuzzy logic can be described as a decision mechanism design [20]. Decision makers can use it to assess vague conditions and, if necessary, examine systems using linguistic data. In 1965, Zadeh [23] developed the fuzzy logic theory and introduced it to address the ambiguity of human judgment. The formulation and resolution of issues that are too intricate or vague to be amenable to conventional methods of analysis are developed by fuzzy set theory [24]. Le Téno and Mareschal [25] were the first to suggest, in 1998, combining fuzzy set theory and the PROMETHEE method and, since then, different studies have combined fuzzy PROMETHEE [24,26,27,28,29,30,31,32,33] to compare, evaluate, and rank alternatives to arrive at a suitable decision based on criteria and assigned weights of importance to each selected criterion. The effective use of PROMETHEE in various medical applications, however, has been demonstrated by recent studies. A study by Duwa et al. [34] applied fuzzy PROMETHEE to compare non-contact temperature reading devices for COVID-19 control. Another study by Ozsahin et al. [35] evaluated cancer treatment alternatives by applying the fuzzy PROMETHEE method and lastly, a medical diagnosis problem was solved by [22] using a proposed Pythagorean fuzzy PROMETHEE method. Thus, the PROMETHEE technique is a method for making decisions based on multiple criteria that compare each alternative pair to each of the chosen criteria. It has the benefits of being simple to use, user-friendly, satisfying in its simplicity, and resolving actual planning issues [35].

In this study, several criteria were proposed, and weights of importance were assigned to each criterion based on expert opinions to evaluate the alternatives. The criteria include prediction accuracy, precision, recall, processing time, sensitivity, and specificity. Each criterion is determined using a linguistic fuzzy scale for usage in PROMETHEE, as shown in Table 1. ML models used for detecting brain tumors were evaluated using the selected criteria, and their importance weights are shown in Table 2. In addition, the Yager index was applied to obtain defuzzification of the fuzzified data. The Yager index is a recommended technique for defuzzification, since it considers all possible points of the sets for this process. Finally, the PROMETHEE approach was implemented using Gaussian preference functions.

It is critical to select the criteria by which alternatives will be evaluated during the decision-making process. Because not all criteria are equally important, weights must be assigned to establish relatively significant levels of each criterion. This means that the most important criteria receive more weight, while the least important criteria receive less weight. By assigning a weight to each criterion, weighting is commonly used to take precedence over important criteria and highlight their relative significance. It is imperative to understand that ranking certain options using the fuzzy PROMETHEE method is based on the applied criteria, alternatives, assigned weights to criteria, and preference functions. Preferred alternatives and criteria may differ from one decision maker to another. This process shows that fuzzy PROMETHEE can be applied in making proper decisions in instances of uncertainty. When the need to select criteria arises, different decision makers may come up with different ideas based on set preferences to compare, evaluate, and rank results. The decision reached by any decision maker will not set a generally accepted standard, as decision makers have different ideas that are unique in their way. To properly rank alternatives, a proper literature search and an expert opinion are needed.

Knowing whether or not a classification/segmentation model can classify brain tumors correctly is critical because it will significantly affect decision-making processes, especially to detect the early onset of tumors in the brain, to lead to effective management and treatment of brain tumor disease. If a model is not accurate and precise enough to detect tumultuous cells in classification and segmentation, a decision maker will not want to start the deployment of the model in order not to risk patients’ lives. A decision maker will also be interested in knowing the number of incorrect predictions a deployed model could generate. When analyzing ML models used in diagnosing brain tumors, some of the most often utilized evaluation measures include prediction accuracy, precision, and specificity. They are the most relevant primary performance metrics for model selection. For this reason, a very high weight was assigned. A model’s ability to recall is another important parameter to be observed in evaluation metrics; hence, the recall criterion is assigned a high weight. The model’s sensitivity to relevant and irrelevant inputs is considered equally important in model deplorability and is, therefore, assigned a high weight. The model’s processing time equally impacts model performance and is important in the selection process of preferred models; thus, the processing time criterion was equally assigned a high weight.

## 3. Results

**Figure 1:**
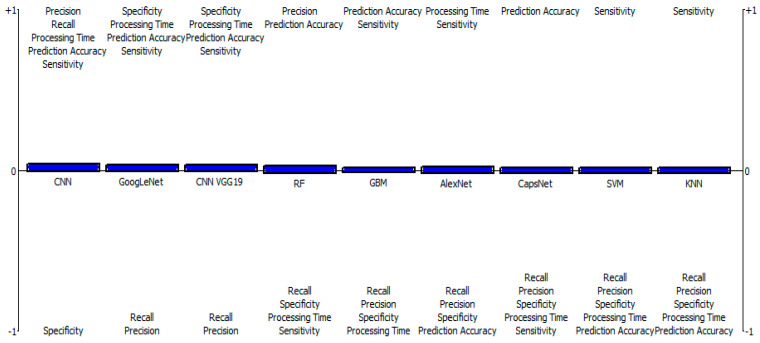
Fuzzy-based PROMETHEE results of positive and negative aspects of ML models.

**Figure 2:**
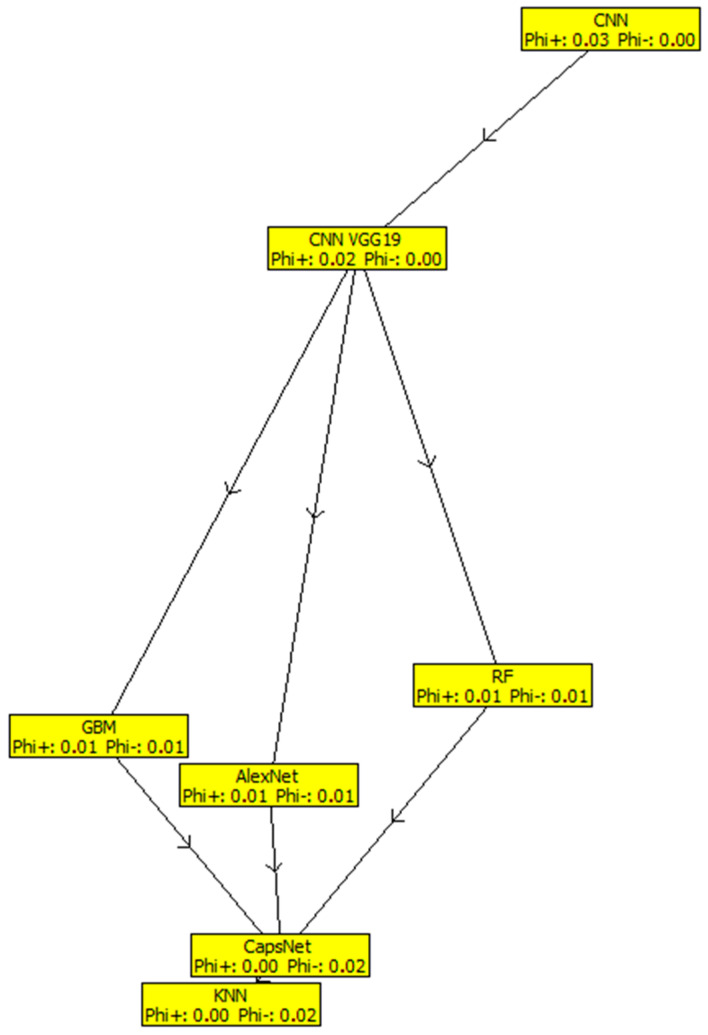
Fuzzy-based PROMETHEE network view.

Table 3 shows the results of the outranking net flows of the considered ML classification/segmentation models for brain tumors by fuzzy-based PROMETHEE model. The results indicate that CNN, with a net flow of 0.0251, stands out in terms of prediction accuracy, precision, recall, specificity, sensitivity, and processing time. This is followed by GoogLeNet with a net flow of 0.0160, and then CNN VGG19, RF, GBM, AlexNet, CapsNet, and SVM models occupied the third, fourth, fifth, sixth, seventh, and eighth positions, with net flows of 0.0160, −0.0022, −0.0043, −0.0063, −0.0134, −0.0154, respectively. The KNN model ranked least among the considered models, with a net flow of −0.0154, due to its low effectiveness across all criteria. Figure 1 shows that CNN has a greater positive value than other models, which include GoogLeNet, CNN VGG19, RF, GBM, AlexNet, CapsNet, and SVM, followed by the KNN model, which comes in last due to its negative properties (below 0 threshold). Meanwhile, Figure 2 shows the fuzzy-based PROMETHEE network view and the efficacy distance of the models, with CNN topping the PROMETHEE network view with the highest positive outranking flow of 0.03, but the lower negative outranking flow of 0.00 with CNN VGG19.

### 3.1. Results Validation

In this section, we conducted a comparative study to determine the efficiency of our proposed methodology with PROMETHEE model and the sensitivity analysis. The goal is to elucidate the level of correctness of our generated results using the proposed method by validating with the sensitivity analysis for our deployed method.

#### 3.1.1. Sensitivity Analysis

The sensitivity analysis examines the effects of the weighted criteria on the ranking results of our evaluated models. The aim of this sensitivity analysis is to identify how changing the weights of the selected criteria affects the stability of our result. When conducting a sensitivity analysis, we normally adjust the weight of one criterion while leaving the weights of the other criteria untouched to see the effects it will have on the ranking outcomes. The importance weights of each parameter in the linguistic scale (see Table 1) were calculated in the current study. The weights of one important criterion, specificity (0.92), would be modified to 0.50 for the purpose of our sensitivity analysis, as indicated in Table 4.

Table 4 displays linguistically articulated weight ranges that specify the criterion limits that can be changed without affecting the final ranking that fuzzy PROMETHEE produced in Table 3. The specificity criterion weight of VH has been changed to M.

The information provided in Table 5 enables us to reach the conclusion that adjustments to the weights of particular criteria, such as specificity with a weight of (VH) but later modified to (M), have no impact on the final ranking outcome. The outranking NetFlow values, positive outranking flow values, and negative outranking flow values all changed; however, the initial complete ranking results remained unchanged. According to the reported results in this study based on simulated parameters, CNN indeed outperforms other ML models taken into consideration in this study, which include GoogLeNet, CNN VGG19, RF, GBM, AlexNet, CapsNet, SVM, and KNN. As a result, we can say that the weights of the chosen criteria have little to no effect on how well ML models for classifying and segmenting brain tumors perform. However, a little variation in NetFlow values was noticed when the weight of one important criterion was changed.

#### 3.1.2. Validation Using Raw Data

In this section, real and raw data in numerical form are simulated using the PROMETHEE program. This is to enable us to verify the possible variations in results when using raw datasets other than linguistic data.

The results presented in Table 6 indicate a slight variation in the overall ranking results of models. The simulation of raw numerical data does not exact significant effects on the overall ranking results of models from the first position to the fifth position of the ranking results. The changes are only observed from the sixth ranking to the ninth ranking. This implies that the CNN model indeed outperformed other considered models in this study for the early detection and classification of brain tumors.

## 4. Discussion

Several supervised learning techniques for the prediction of brain tumor cancer have been developed and comparisons have been made in the existing literature. The result of this present study is consistent and similar to the results of the following studies: Brown et al. [38] presented a machine learning approach for the analysis of the unstructured text of patients’ image demographics from MRI scanning. The ML algorithm interpreted and chose the most appropriate MRI brain imaging sequence with clinical significance. They compared three ML models, support vector machine (SVM), random forest (RF), and gradient-boosting machine, to a baseline model. Their aim was to assess and compare ML models based on precision, accuracy, recall, and Hamming loss. The results from the study showed that the gradient-boosting ML model outperformed the radiologist sequence choice, which was the baseline model, and demonstrated efficiency among the three models with an accuracy of 95%, precision of 86%, recall of 80%, and 0.0487 of Hamming loss. In another study by Tandel et al. [39], a convolutional neural network (CNN) was used to design a high-performance transfer-learning-based model for grading and classifying brain tumors through MRI data. The study compared a deep learning model with six ML models based on performance parameters (AUC, recall, precision, F-score, and accuracy). The transfer-learning-based CNN model was compared to another six different ML models, namely: support vector machine, K-nearest neighbor, naive Bayes, decision tree, linear discrimination, and ensemble. The study results proved that the CNN-based deep learning model outperformed the six ML models in the classification of multiclass tumor datasets, as follows: accuracy 100%, precision 100%, recall 100%, F-score 100%, and AUC = 1, and the overall mean performance parameters for ML models are as follows: accuracy 97%, precision 99.46%, recall 99.48%, F-score 99.38%, and AUC = 1.

A study by Yang et al. [40], demonstrated the utility significance of deep learning with transfer learning models to preoperatively classify lower-grade gliomas and higher-grade gliomas of brain tumors using MRI images. The classification was performed on patients using five-fold cross-validation. The effects of transfer learning were assessed on two CNN models (AlexNet and GoogLeNet). The performance of the two models was evaluated by assessing the training loss, validation loss, validation accuracy, test accuracy, and test AUC. GoogLeNet is a more complex and deeper CNN model because of its new module called “inception”. AlexNet had a significantly improved performance over other non-deep-learning methods in terms of visual recognition. But when compared to GoogLeNet, GoogLeNet outperformed AlexNet, even with its lesser inception structure, with a mean value validation accuracy of 0.867, test accuracy of 0.909, and test AUC of 0.939. Meanwhile, the AlexNet mean value validation accuracy was 0.866, test accuracy was 0.855, and test AUC was 0.895. Another study by Sekhar et al. [42] trained a CNN model, i.e., GoogLeNet, to classify brain tumors into three classes: pituitary, meningioma, and glioma. The experimental results compared GoogLeNet with other existing models and found the GoogLeNet model to be a superior model in terms of performance measures such as accuracy, specificity, etc.

Swatı et al. [43] focused on the classification of a multiclass brain tumor for MRI using transfer learning pre-trained CNN models. In this study, a block-wise fine-tuning strategy was adopted for an enhanced classification result. The datasets used in the study were involving cases of glioma, meningioma, and pituitary brain tumors, which are the three types of brain tumors. The five-fold cross-validation test was adopted for the evaluation to ensure a robust study methodology. The proposed method was assessed on a T1-weighted contrast-enhance MRI (CE-MRI) dataset, which is usually a small dataset compared to natural image datasets. A pre-trained CNN VGG19 trained on a large dataset of ImageNet was used. The CNN VGG19 has sixteen convolutional layers with three other connected layers. The pre-trained VGG19 was used to initialize the weights of CNN and a block-wise fine-tuning of the CNN VGG19 was proposed for the enhancement of the classification results and to overcome overfitting. Classification performance was evaluated based on recall, precision, F1 score, and accuracy. The result indicated that the CNN VGG19 model performed best when compared to state-of-the-art classification. A comparison was also made between the CNN VGG19 and the other three pre-trained models (i.e., VGG16, AlexNet, and VGG19). The VGG16 and AlexNet achieved an average classification accuracy of 89.95% and 94.65%, respectively, but the CNN VGG19 outperformed them with a five-fold classification accuracy of 94.82%.

A study by Vijayakumar et al. [44] compared the capsule neural network against the neural network model (NN), convolutional neural network (CNN), and ResNet. Capsule neural network (CapsNet) is a machine learning method that can train a lesser number of datasets when compared to CNN. The study aimed to compare CapsNet with NN, CNN, and ResNet, based on the proposed method of classifying brain cancer from its early stage and narrowing the basis of comparison to the following parameters: training accuracy, testing accuracy, and prediction time. The results from the study proved that CapsNet had a better classification performance than NN, CNN, and ResNet models, followed by the CNN model.

Maharjan et al. [41] presented a multiclass classification study for brain tumors to avoid the possibility of overfitting data in order to improve classification accuracy. The comparison was based on performance evaluation metrics such as accuracy and processing time. In the proposed CNN-based solution, the authors used a modified softmax loss function, claiming a 2% improvement in accuracy and an additional 40–50 ms improvement in speed over previous approaches.

Jena et al. [36] proposed several classification and segmentation models for brain tumors, namely: support vector machine (SVM), K-nearest neighbors (KNNs), binary decision tree (BDTs), random forest (RF), fuzzy C-means (FCM), Kmeans, and ensemble methods. The results from the experiment indicated the efficacies of the models, with performance accuracies of SVM 94.25%, KNN 87.88%, BDT 89.57%, RF 98.99%, and ensemble methods 97%, respectively. Another study by Deepak et al. [37] adopted the CNN and SVM algorithms for the classification of brain tumors. An MRI image *Figshare* dataset was used to evaluate the fully automated system, as the CNN extracts features from brain MRI images. The SVM was used in combination with the CNN features. A five-fold cross-validation procedure for testing and evaluation was implemented, and the results indicated that the proposed model attained a superior performance accuracy of 95.82%, better than other state-of-the-art models.

The CNN model has been observed, in this present study, to be a superior model for the early detection and classification of brain tumors. This is consistent with the results of the literature cited above. However, the concept of validation of model performance in this study is different from the usually deployed validation metrics that have always been based on performance evaluation metrics such as accuracy, F1 score, log loss, precision, recall, AUC, specificity, and so on. Our MCDM evaluation makes use of a number of chosen weighted validation metrics to evaluate the efficacy of models. Accuracy, precision, recall, F1-score, log loss, etc., are commonly used evaluation metrics, but recently decision makers have sought a more robust method of incorporating additional metrics into the model selection process. These are the results of the study. This research investigates the use of fuzzy PROMETHEE as an alternative approach to evaluating performance metrics that are utilized in the process of selecting ML models.

## 5. Limitations of This Study

In most MCDM systems, the assignment of criteria weights and the ranking of alternatives provide fundamental challenges. In our situation, the ML models for the diagnosis of brain tumors are typically prone to ongoing development, making it difficult to deliver the proper score of alternatives. In other words, other researchers might add extra steps or activities, or even combine different models, which could improve the performance of models. Since different models are utilized in ways that might not be fully understood or agreed upon due to conflicting results in the literature, there is uncertainty and a lack of consistency with the scoring of alternatives. In this study, we used a thorough and filtered assessment of the literature to base our comparison and interviewed subject-matter experts for their professional contributions.

The way the criteria are weighted is another challenging factor that makes decision making more subjective and ambiguous. The PROMETHEE methodology does not have a predetermined process or mechanism for deciding how much weight to give each criterion. However, the importance of each criterion was determined based on fuzzy logic process. To get around this problem, a sensitivity analysis is required at the very end of the decision-making process.

## 6. Conclusions and Recommendations

By comparing nine widely used models (support vector machine (SVM), random forest (RF), gradient-boosting model (GBM), convolutional neural network (CNN), K-nearest neighbor (KNN), CNN VGG19, AlexNet, GoogLeNet, and CapsNet) using the fuzzy PROMETHEE method, this paper illustrates the significance of ML models used for the classification of brain tumors. We observed that CNN had greater predicting accuracy, precision, recall, processing time, sensitivity, and specificity when comparing the classification models. The robustness of the fuzzy PROMETHEE model was evaluated via a sensitivity analysis because one synthesis approach cannot always address all decision-making issues.

Future research can enhance this work by incorporating more models and, more crucially, weighted factors that are related to model selection. A variety of decision-making models and algorithm combinations, which were excluded from this study, can be utilized to compare and contrast models in order to improve computational outcomes and spot differences in outcomes. The findings of this study indicate that the fuzzy PROMETHEE approach can be used to select models and is a reliable decision-making tool.

## Figures and Tables

**Table 1:** Linguistic fuzzy scale.

Ranking Linguistic Scale	Fuzzification Scale/Weighting
VH	(0.75, 1, 1)
H	(0.50, 0.75, 1)
M	(0.25, 0.50, 0.75)
L	(0, 0.25, 0.50)
VL	(0, 0, 0.25)

Very High = VH, High = H, Medium = M, Low = L, Very Low = VL.

**Table 2:** Dataset for evaluating ML models used for brain cancer diagnosis.

Aim/Importance Weights	Max/VH	Max/VH	Max/H	Min/H	Max/H	Max/VH
Alternatives/Criteria	Average Prediction Accuracy	Average Precision	Average Recall	Average Processing Time in secs	Average Sensitivity	Average Specificity
Support Vector Machine (SVM) [36,37]	(94.1) H	(90.9) H	(91) H	(429) M	(95.02) H	(93.99) H
Random Forest (RF) [36,37]	(96.4) VH	(96) VH	(90) H	(412) M	(89.89) M	(95.23) H
Gradient-boosting Model (GBM) [38]	(96) VH	(90) H	(90) H	(405) M	(90.10) H	(88.21) M
Convolutional Neural Network (CNN) [39]	(99.9) VH	(100) VH	(99) VH	(301) L	(95) H	(98.13) VH
K-nearest neighbor (KNN) [36,37]	(90.88) H	(90.11) H	(90) H	(381) M	(90.16) H	(90.12) H
AlexNet [40,41]	(86) M	(84) M	(89) M	(348) L	(90.09) H	(90.8) H
GoogLeNet [40,41,42]	(98) VH	(95.78) H	(94.10) H	(322) L	(94.8) H	(97.12) VH
CNN VGG19 [43]	(99) VH	(90.5) H	(99.10) VH	(311) L	(95.2) H	(94.69) VH
CapsNet [41,44]	(96.56) VH	(84.61) M	(86) M	(372) M	(81.01) M	(90.10) H

70–79 (L), 80–89 (M), 90–95 (H), 95–100 (VH).

**Table 3:** Fuzzy-based PROMETHEE results/preference table (support vector machine (SVM), random forest (RF), gradient-boosting model (GBM), convolutional neural network (CNN), K-nearest neighbor (KNN)).

Rank	ML Models	Net Flow	Phi+ Flow	Phi− Flow
1	CNN	0.0251	0.0276	0.0025
2	GoogLeNet	0.0160	0.0195	0.0035
3	CNN VGG19	0.0160	0.0195	0.0035
4	RF	−0.0022	0.0124	0.0146
5	GBM	−0.0043	0.0057	0.0100
6	AlexNet	−0.0063	0.0071	0.0134
7	CapsNet	−0.0134	0.0037	0.0171
8	SVM	−0.0154	0.0020	0.0175
9	KNN	−0.0154	0.0020	0.0175

**Table 4:** Preference criteria for sensitivity analysis.

Weight	VH	VH	H	H	H	M
Criteria	Prediction Accuracy	Precision	Recall	Processing Time	Sensitivity	Specificity

**Table 5:** Sensitivity analysis results with fuzzy PROMETHEE (support vector machine (SVM), random forest (RF), gradient-boosting model (GBM), convolutional neural network (CNN), K-nearest neighbor (KNN).

Rank	ML Models	NetFlow	Phi+ Flow	Phi− Flow
1	CNN	0.0286	0.0301	0.0015
2	GoogLeNet	0.0131	0.0169	0.0038
2	CNN VGG19	0.0131	0.0169	0.0038
4	RF	−0.0012	0.0135	0.0147
5	GBM	−0.0034	0.0063	0.0097
6	AlexNet	−0.0057	0.0077	0.0134
7	CapsNet	−0.0134	0.0041	0.0174
8	SVM	−0.0156	0.0022	0.0178
8	KNN	−0.0156	0.0022	0.0178

**Table 6:** Validation results with fuzzy PROMETHEE.

Rank	ML Models	Outranking Net Flow	Phi+	Phi−
1	CNN	0.7433	0.7434	0.0001
2	GoogLeNet	0.4653	0.5519	0.0866
3	CNN VGG19	0.4285	0.5257	0.0973
4	RF	0.0082	0.2927	0.2844
5	GBM	−0.1164	0.2267	0.3432
6	SVM	−0.2805	0.1497	0.4301
7	KNN	−0.3360	0.1466	0.4826
8	AlexNet	−0.4504	0.1278	0.5782
9	CapsNet	−0.4620	0.1283	0.5903

## Data Availability

Data is available upon the requests from the authors.
